# Inherited Sensory and Autonomic Neuropathy in a Border Collie, Interest of Oclacitinib for the Control of Self-Mutilation

**DOI:** 10.3390/vetsci9030127

**Published:** 2022-03-10

**Authors:** Caroline Leonard, Iris Van Soens, Jacques Fontaine

**Affiliations:** Department for Clinical Sciences, Faculté de Médecine Vétérinaire, University of Liege, Quartier Vallée 2, Avenue de Cureghem 3, Sart-Tilman, 4000 Liege, Belgium; iris.vansoens@uliege.be (I.V.S.); jacques.fontaine@uliege.be (J.F.)

**Keywords:** canine, *FAM134B* gene, neuropathies, genetic, treatment

## Abstract

Sensory and autonomic neuropathy was diagnosed in a five-month-old Border Collie puppy, who presented with progressive self-mutilation, proprioceptive ataxia and urinary incontinence. In the Border Collie, sensory neuropathy is different from what is observed in acral mutilation syndrome, as the genetic mutation is linked to an inversion disrupting the *FAM134B* gene. Diagnosis was based on history, clinical signs and genetic testing. The prognosis of sensory neuropathies is poor and no curative treatment is available. In the present case, oclacitinib was started for symptomatic treatment of the self-mutilation. A good control of the self-mutilation was quickly observed with an improvement in quality of life for five months. Unfortunately, progression of neurological signs with severe proprioceptive deficits, ataxia, muscular atrophy and urinary/fecal incontinence was observed. Five months after diagnosis, the owner elected for euthanasia.

## 1. Introduction

Inherited sensory neuropathies are rarely reported in canine diseases. In dermatology, the most frequently encountered canine sensory neuropathies are the numerous publications considering the AMS in German short-haired pointers, English pointers, English springer spaniels, French spaniels or miniature pinschers [1,2,3,4,5,6,7]. The disease is observed in the early life, progressing slowly to a severe self-mutilation of the distal extremities of limbs [8]. The prognosis is always poor because of the dog’s bad quality of life. To date, no recommended treatment can be advised. The case here reported refers to another sensory neuropathy less commonly observed by dermatologists but probably more frequently recognized by neurologists.

## 2. Case Presentation

**Figure 1:**
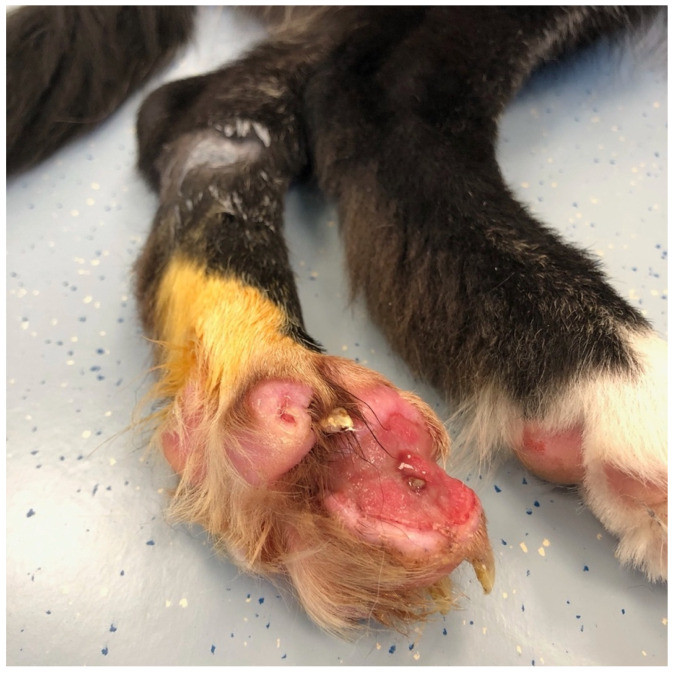
Large wounds on the lateral part of digit II and III of the left hind limb.

A five-month-old female Border Collie was presented to the dermatology department for evaluation of a severe digital wound of one month duration. Previous treatments with Povidone-Iodine (Iso-betadine^®^; Mylan, Hoeilaart, Belgium) and healing ointment (unknown) gave no improvement. Intense licking, chewing and biting of the feet leading to self-mutilation was reported by the owner. On physical examination, large wounds were present on the lateral part of digit II and III of the left hind limb (Figure 1). The lesions seemed unsensitive to a painful stimulus. General and neurological examination did not reveal other abnormalities at that time.

The problem of the dog was defined as digital pruritic or self-traumatic ulcerative dermatitis. Differential diagnoses included a vascular disease (arteriovenous fistula/shunt), traumatic injury or a genetic defect such as acral mutilation syndrome (AMS). Due to the early onset of signs and the suspicion of self-mutilation rather than pruritis, AMS was considered even though the Border Collie is not a predisposed breed to this disease. Cytological examination by impression smear of the ulcerative lesion showed neutrophils and admixed extracellular bacteria, suspected to be of oral origin. A genetic test for acral mutilation syndrome (looking for the mutation of the Glial-cell = derived neurotrophic factor gene, *GDNF*) was performed (Synlab veterinary, Heppignies, Belgium). Pending the genetic result, the dog was treated with a cream containing lidocaine and prilocaine (Emla^®^ 5% cream; Aspen, Rueil-Malmaison, France) and pregabalin at 2.6 mg/kg twice daily (Lyrica^®^; Pfizer, Paris, France) for one month. The test for AMS came back as homozygous normal. One month after treatment initiation, new lesions were observed on the digital extremities of both front limbs. On the left hind limb, digit III was swollen, the skin ulcerated and necrotic and digit II was also partially necrotic. The nails were intact. On the left front limb, the medial aspect of digit II presented a crusting lesion. On the right front limb, digit II was also affected with presence of crusts and early necrosis. During observation, the dog showed intense licking and biting on all limbs, even those without lesions. At neurological examination, the dog presented a normal gait with reduced postural reactions on both hind limbs and the right front limb. The withdrawal reflex was absent on all limbs and nociception was severely reduced to absent on all limbs. The rest of the neurological examination was normal. Owing to the progressive evolution of the lesions, the abnormal findings on neurological examination and the poor response to medical treatment, another sensory canine neuropathy, reported in the Border Collie, was suspected and a test looking for a mutation in the *FAM134B* gene was requested (Laboklin GMBH & Co., Bad Kissingen, Germany). The dog was homozygous mutated. A diagnosis of inherited sensory and autonomic neuropathy of the Border Collie associated with an inversion disrupting the *FAM134B* gene was concluded.

As no clinical improvement was observed with oral pregabalin and lidocaine/prilocaine cream, a trial with oclacitinib (Apoquel^®^, Zoetis, Belgium) at 0.57 mg/kg orally twice daily was proposed to the owner, even though the long-term prognosis for the disease was very poor. A quick improvement in the signs of self-mutilation was observed. The dog was comfortable and did not lick or bite his extremities.

**Figure 2:**
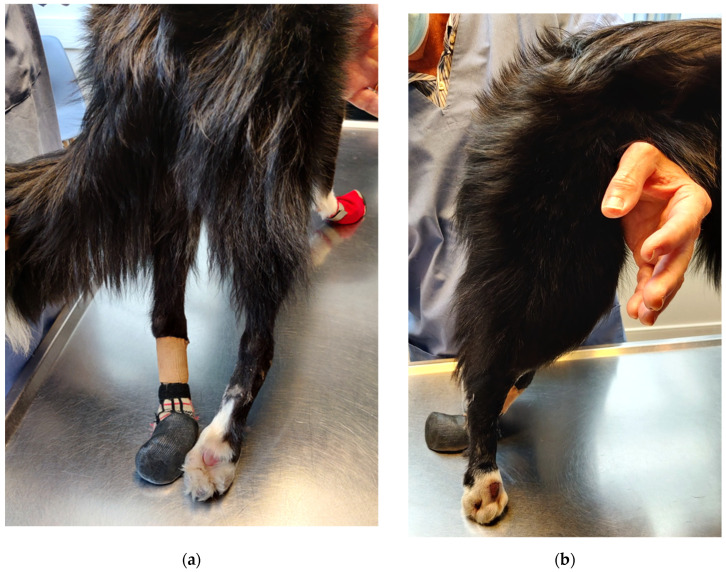
(**a**) Severe proprioceptive deficits with spontaneous knuckling and hyperextension of the tarsal joint. (**b**) Absence of self-mutilation with improvement of the digit lesions with oclacitinib administration.

At the age of ten months, a new consultation was requested because of progressive neurological motor deficits and urinary/fecal incontinence. Despite a good improvement of the self-mutilation with oclacitinib administration, and the use of protective footwear to avoid injuries, the dog presented severe proprioceptive deficits of both hind limbs with spontaneous knuckling and hyperextension of the tarsal joints (Figure 2). Generalized muscle atrophy was observed and was more pronounced on the hind limbs. Due to the progressive neurological deficits and the poor prognosis, making the dog’s quality of life poor, the owner elected to euthanize the dog. Necropsy was declined by the owner.

## 3. Discussion

In humans, inherited peripheral neuropathies are classified into two groups, hereditary sensory neuropathies (HSNs) and hereditary sensory and autonomic neuropathies (HSANs), according to the phenotype and the genetic variants. Up to date, twenty genes have been discovered and identified to be implicated in those diseases. Clinical features are depending on the localization of the mutation, as each gene has a precise role in the nervous system [8].

Based on the human classification, Granger and colleagues have proposed a canine classification of neuropathies into different groups: sporadic motor and sensory neuropathies (SMSN), inherited motor and sensory neuropathies (IMSN) and inherited sensory and autonomic neuropathies (ISAN) [9]. Canine IMSN are more frequently reported than ISAN, as is also noted in humans. A unique genetic origin is observed for each disease subtype and is correlated with a specific at-risk breed [9]. The responsible genetic mutation involved in ISAN has been discovered in some breeds. The lincRNA upstream of the *GDNF* gene was described in German shorthaired pointer, English springer spaniel and French spaniel dogs and inversion disrupting of *FAM134B* gene in Border Collies and their crosses [6,10,11]. For some breeds affected with the ISAN, the causative genetic mutation has not been discovered. A human form homologue of the sensory neuropathy of the Border Collie is known and called HSAN2B, where a mutation of the *FAM134B*/*RETREG1* gene is also described [12]. This gene is responsible for encoding a cis-Golgi protein, present in sensory and autonomic neurons [10,12]. In both human and canine forms, an autosomal recessive trait is described [10,13].

In the present case, the genetic mutation is linked to an inversion disrupting the *FAM134B* gene, as was reported by similar case reports in the literature [10,11]. The genetic abnormality induces signs of progressive proprioceptive ataxia associated with acral self-mutilation, affecting mostly the pelvic limbs, and in some cases autonomic disorders characterized by urinary incontinence and regurgitation [10,14,15]. The loss of proprioception and nociception is caused by axonal degeneration, endoneural fibrosis and extensive large nerve fiber loss [14,15]. The onset of clinical signs is generally noticed between the age of two to seven months old [10,14,15,16]. At the initial presentation of the dog in this case, AMS could not be excluded based on the initial clinical signs (loss of pain perception), even though the disease had not been previously reported in the Border Collie. However, with the progressive neurological deficits and the apparition of urinary incontinence, the clinical differentiation between AMS and sensory neuropathy of the Border Collie was more obvious. In AMS, no motor, autonomic and proprioceptive neurological deficits are observed, and also normal spinal reflexes are appreciated [1,2,3,17].

The treatment of sensory neuropathies in Border Collie is only based on early care (bandage and Elizabethan collar) for prevention of injuries and self-mutilations [4,17]. In the present case, due to the lack of response to pregabalin, oclacitinib was tried and a quick improvement was observed. To the author’s knowledge, this is the first report of sensory neuropathy managed with oclacitinib allowing control of the self-mutilation. Oclacitinib, by its inhibition on Janus kinase family members and cytokines, is well known to have an effect on the pruritic pathway [18]. The possible effect on other neurological pathways, indirectly inducing the self-mutilation reaction, is unreported. Unfortunately, due to lack of effect of oclacitinib on disease progression, its use is only limited to improve the quality of life. As it is a progressive disease, affecting the proprioception, nociception but also autonomic systems, euthanasia is often elected for by the owner within 2 years of diagnosis.

## 4. Conclusions

In conclusion, this case report highlights the importance in the selection of genetic testing. The promising effect of oclacitinib as a treatment improving quality of life in sensory neuropathy in Border Collies should be confirmed in other cases or in other sensory neuropathies such as AMS.

## Data Availability

Not applicable.
